# Ultrasound-Guided Percutaneous Needle Electrolysis Versus Surgery for Carpal Tunnel Syndrome: A Randomized Clinical Trial

**DOI:** 10.3390/healthcare14040507

**Published:** 2026-02-16

**Authors:** Fermín Valera-Garrido, Jesús Segura-León, Paula García-Bermejo, Francesc Medina-Mirapeix

**Affiliations:** 1 MVClinic Institute, 28600 Madrid, Spain; ferminvalera@mvclinic.es; 2 Campus of Montepríncipe, CEU San Pablo University, 28668 Madrid, Spain; 3 Invasive Physiotherapy Department, Getafe Football Club, 28903 Madrid, Spain; 4 CLIFIS Center, 13170 Ciudad Real, Spain; 5 Department of Physiotherapy, Faculty of Medicine, Health and Sports, European University of Madrid, 28670 Madrid, Spain; paula.garcia2@universidadeuropea.es; 6 DINAMIA Center, 28805 Madrid, Spain; 7 Department of Physiotherapy, Health Sciences Campus, University of Murcia, 30120 Murcia, Spain; mirapeix@um.es

**Keywords:** carpal tunnel syndrome, median nerve, open carpal tunnel release, surgery, percutaneous needle electrolysis, Boston Carpal Tunnel Questionnaire

## Abstract

**Background/Objectives**: Carpal tunnel syndrome (CTS) is the most common entrapment neuropathy of the upper limb. The aim of this study was to analyze the safety and effectiveness of ultrasound-guided percutaneous needle electrolysis (PNE) and open carpal tunnel release (OCTR) in patients with moderate-to-severe CTS. **Methods**: A total of 185 patients with idiopathic CTS were assigned to either the electrolysis group (75 patients) or the surgery group (73 patients); 112 patients completed the final follow-up assessment 12 months after randomization. The surgical procedure consisted of OCTR. The electrolysis group received four sessions of US-guided PNE applied every seven days. Main outcomes were nights waking up due, pain, paresthesia, Boston Carpal Tunnel Questionnaire Symptom Severity Scale (BCTQ-SSS), Functional Status Scale (BCTQ FSS) and adverse events. These variables were evaluated in the short (6 weeks), medium (3 months), and long term (6 and 12 months). **Results**: In the short term (6 weeks), both interventions did not show significant differences in the severity of symptoms; however, the electrolysis group had less adverse events than the surgery group (2 vs. 100). In the medium (3 months) and long term (6 and 12 months), surgery was slightly more effective regarding nocturnal awakenings, paresthesia and BCTQ-SSS (*p* < 0.002). **Conclusions**: US-guided PNE may be a safe and effective technique for patients with moderate-to-severe CTS with a sustained long-term pattern of improvement. Although both treatments were effective, OCTR showed superior long-term symptom reduction. Therefore, PNE may serve as a first-line or bridging treatment in selected clinical scenarios.

## 1. Introduction

Carpal tunnel syndrome (CTS) is the most common compressive neuropathy of the upper extremity [1,2]. It results from compression of the median nerve as it passes through the carpal tunnel in the wrist [3], causing pain, numbness, and tingling in the hand and fingers [3]. Risk factors include diabetes mellitus, hypothyroidism, advanced age, obesity, female sex, pregnancy, rheumatoid arthritis, and repetitive wrist movements, among others [4,5].

The recent Cochrane review in 2024 establishes the state of the art on surgical versus conservative treatments for carpal tunnel syndrome [6]. At short-term follow-up (up to 3 months), surgical and conservative treatments are useful to improve symptoms in patients with CTS [6]. Surgery does not provide clinically important benefits compared to various conservative treatments. At long-term follow-up (6 and 12 months), the pattern of improvement of conservative treatments (e.g., splinting and manual therapy) appears to slightly decrease over time, and thus surgery provides better clinical improvement. However, despite these advantages, the Cochrane review advises against surgery for all patients, as it offers only modest benefits and carries potential risks like persistent pain or hypertrophic scarring at the wrist incision site [6]. In this situation, surgical procedures should be intended for severe cases or for patients with unsatisfactory results after conservative treatment [7,8].

Recently, there has been growing interest in minimally invasive procedures within healthcare. In CTS, the development of endoscopic or ultrasound-guided surgical procedures has improved the effectiveness and safety of treatment in the short term [9]. Other techniques such as percutaneous needle electrolysis (PNE), which involves applying a galvanic current through a needle under ultrasound guidance [10,11,12], have shown promising potential. Animal models have demonstrated PNE’s ability to reverse symptoms associated with nerve entrapment [13], and human case series have shown promising results [14] and few adverse events [15].

The objective of this study was to compare the safety and effectiveness of surgical release and PNE on the functional status and symptoms of patients with moderate-to-severe carpal tunnel syndrome. Specifically, the study aimed to: (1) determine the effectiveness and adverse events in the short term; and (2) analyze the pattern of symptom improvement in the medium and long term.

## 2. Materials and Methods

### 2.1. Study Design

A randomized clinical trial was conducted at the General Traumatology Department of the Hospital General Universitario of Ciudad Real (Spain), comparing the safety and efficacy of US-guided PNE and surgical intervention in patients with CTS. The study protocol was approved by the Institution’s Ethics Committee (Minute No. 01/2014), prospectively registered at https://www.clinicaltrials.gov/ (registration number NCT04216147) and designed following the CONSORT statement guidelines. Enrolled patients provided informed consent and confirmed willingness to receive US-guided PNE or OCTR before study participation. Eligible patients were randomized to receive US-guided PNE or OCTR and remained in follow-up for 1 year.

### 2.2. Participants

All patients with clinically suspected CTS were examined for eligibility to participate in the study. The inclusion criteria were: (1) diagnosis of moderate-to-severe CTS based on electrodiagnostic studies [16,17], together with symptomatic characteristics, following the criteria established by Harrington et al. [18] and (2) age 18 years or older. The exclusion criteria were: (1) unable to provide accurate or quality information due to the inability to read texts because of illiteracy, blindness, or language limitations; (2) cognitive impairment; (3) subjects who could provide biased information; (4) those with a history of treatment of CTS with wrist surgery or infiltrations in the previous three months; (5) a history of wrist trauma or surgery; (6) a history of potential concurrent cause to idiopathic CTS (such as diabetes, hyperthyroidism, chronic rheumatoid arthritis, renal failure with hemodialysis, pregnancy or lactation); or (7) the existence of diffuse peripheral neuropathy or cervical radiculopathy.

### 2.3. Randomization and Blinding

The random allocation schedule was performed using permuted blocks of six patients using random tables. The sequence was prepared by a researcher not involved in either the initial eligibility screening or patient assignment. This same researcher prepared and coded opaque envelopes containing the allocation sequence. After the initial screening, patients were referred to an administrative research assistant, who opened the next allocation envelope. Thus, the initial screening provider remained blinded to the allocation sequence. After opening the envelope, appointments were generated for the assigned intervention.

### 2.4. Interventions

#### 2.4.1. US-Guided Percutaneous Needle Electrolysis (PNE)

US-guided PNE was applied using a specific device (EPI^®^ s.c.p. Sant Quirze de Valles, Barcelona 08192, Spain), which produces a continuous galvanic current through the cathode (modified electrosurgical scalpel with the needle) while the patient holds the anode (handheld electrode). A portable General Electric^®^ Logic-E ultrasound device was used with a 12 L-RS linear probe. Ultrasound changes in the median nerve have been previously described [19].

**Figure 1:**
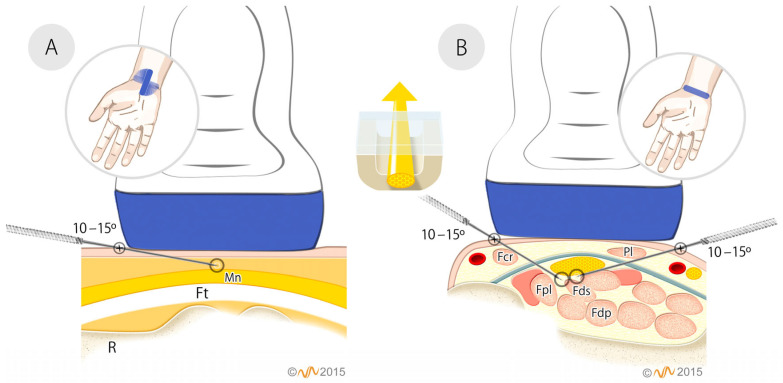
US-guided PNE according to the protocol by Valera and Minaya [20]. (**A**) Approach to the superficial interface. Longitudinal view of the median nerve, in-plane approach from proximal to distal at 10–15°. Mn: Median nerve. Ft: Flexor tendon. R: Radius. (**B**) Approach to the deep interface. Transverse view of the median nerve, in-plane approach from radial to ulnar or from ulnar to radial, depending on the area of nerve compression, at an angle of 10–15°. Fcr: Flexor carpi radialis. Pl: Palmaris longus. TCL: Transverse carpal ligament (gray line). Fpl: Flexor policis longus. Fds: Flexor digitorum superficialis. Fdp: Flexor digitorum profundus. The blue line on the wrist indicates the position of the probe. (**A**) It begins with a transverse view of the median nerve, followed by a longitudinal view to confirm the location of the median nerve. (**B**) Transverse view. The central schematic with the yellow upward arrow represents the median nerve within the carpal tunnel, highlighting its anatomical relationship with the surrounding structures. The transparent, dome-shaped structure above it represents the TCL. Its semi-transparent appearance allows visualization of the nerve beneath it and emphasizes its role as the roof of the tunnel. The brown structure at the base represents the carpal bones, which form the rigid floor and lateral walls of the carpal tunnel.

During US-guided PNE, the participants were placed in the supine position with their forearm resting on the table. Chlorhexidine Alcoholic Transparent (Lainco^®^ 2%, 08191 Rubí, Barcelona, Spain) was used for the disinfection of healthy skin. Subsequently, an acupuncture needle measuring 0.30 mm × 30 mm (Physio Invasiva^®^ needles, PRIM Physio, 28938, Madrid, Spain) was inserted, using a long axis approach, into to the deep interface between the median nerve and the superficial flexors of the fingers and the flexor pollicis longus (first approach), as well as to the superficial interface between the median nerve and the transverse carpal ligament (TCL) (second approach) (Figure 1) [20].

The US-guided PNE was performed using an intensity (I) of 1.5 mA, for 3 s (time) (T) and 3 impacts (I) (1.5:3:3) (I:T:I), following the protocol by Valera and Minaya [20]. Participants in the treatment group received four sessions of US-guided PNE, applied every seven days [20]. The PNE procedure was performed by a physiotherapist with over 10 years of experience in ultrasound evaluation and interventional techniques.

#### 2.4.2. Surgery

The procedure consisted of an open carpal tunnel release (OCTR) performed through a standard longitudinal palmar incision along the axis of the ring finger, between the thenar and hypothenar eminences. The incision extended distally to allow dissection of the palmar fascia with a surgical blade for direct visualization of the TCL, median nerve, its branches, and the superficial palmar arterial arch. The underlying TCL was then divided longitudinally along its ulnar aspect. The incisions were closed with sutures. Detailed procedural steps have been previously reported [21]. The surgical technique was carried out by a surgeon with over 10 years of experience.

Patients in both groups did not receive subsequent conservative treatment during follow-up.

### 2.5. Outcome Measurements

All assessments were performed by a single investigator who was blinded to allocation and treatment details. Baseline data and other characteristics such as hand dominance, symptomatic side, duration of symptoms, and presence of nocturnal symptoms were collected at the baseline (Table 1). Assessments were conducted before the intervention and at 6 weeks, 3 months, 6 months, and 12 months after the intervention.

#### 2.5.1. Primary Outcomes

The main primary outcome measure was the mean number of nights the patient awoke due to symptoms during the past week from baseline over the course of four follow-up visits. Other primary outcomes were mean severity of pain and paresthesia, measured on an 11-point numerical rating scale (0 = “no symptoms”, 10 = “very severe symptoms”) and their multidimensional score using the Boston Carpal Tunnel Questionnaire-Symptom Severity Scale (BCTQ-SSS) which consists of 11 questions about symptoms experienced over the past two weeks (scored from 1 = mildest to 5 = most severe). For safety, the total number of adverse events (mild–moderate and severe–very severe) per patient was recorded [22].

#### 2.5.2. Secondary Outcomes

The patient’s functional status was assessed using the Boston Carpal Tunnel Functional Status Scale (BCTQ FSS) [23], which includes 8 items evaluating difficulty in performing various activities of daily living during the past two weeks. Each item is scored from 1 (no difficulty) to 5 (cannot perform the activity at all).

### 2.6. Statistical Analysis

Categorical variables are presented as number (and percentage), while continuous variables are reported as means (and standard deviation) or medians (and interquartile ranges). Normal distribution (Shapiro–Wilk test) and homoscedasticity (Levene test) of the data were verified before groups were compared at baseline. We compared baseline characteristics between those patients who completed the study and those who withdrew, and between withdrawals from both groups as well, because there was differential attrition between groups. Finally, we used multiple logistic regression analyses to examine whether withdrawals were associated with baseline characteristics.

We did not impute missing data and, thus, only available data was used for the analysis of each specific variable. We also do not impute risks attrition bias because, from the analyses between withdrawals from both groups, we assumed that similar participants leave from the two groups (i.e., there low bias risk). All statistical analyses were conducted using a statistical software package (IBM SPSS Statistics for Windows, version 25.0, Armonk, NY, USA) with α = 0.05.

For the safety objective (short-term, 6 weeks), the total number of adverse events per patient and the incidence rate ratios (IRRs) between the treatment groups were calculated. For the effectiveness objective, two-way mixed ANOVAs were conducted to determine the significance of group effect (between subjects), time effect (within subjects) and interaction effect (group_time). Partial eta squared (ηp^2^) was used for the effect sizes, and absolute between-group differences with confidence intervals were calculated. Significant interaction effects were plotted to facilitate interpretation. Additionally, because of baseline imbalance multiple variable analyses (linear regression) were performed to adjust the group effect for the influence of eventual differences between the groups at baseline in prognostic indicators (dominant-hand involvement and baseline pain). For these analyses, all patients who withdrew from the study were excluded. While we assumed low bias risk from baseline comparisons, we performed additional sensitivity analyses on the main primary outcome to assess the robustness of results considering duration of current episode or functional status as covariates.

The sample size calculation was based on ensuring sufficient precision for the planned two-way mixed ANOVA with 5 measurements (from baseline to 12 months). An initial sample of 53 patients per group, or a total of 106 patients, would provide a power of at least 0.9, assuming an alpha level of 0.05 and effect size of 0.15 (near-to-small) for testing the hypothesis of whether there was a time intervention interaction regarding the number of nights waking up. Based on previous experiences with similar research projects, we assumed a maximum attrition rate of 30% for the one-year-long study. Therefore, we needed a total of 138 patients to achieve the desired power.

## 3. Results

**Figure 2:**
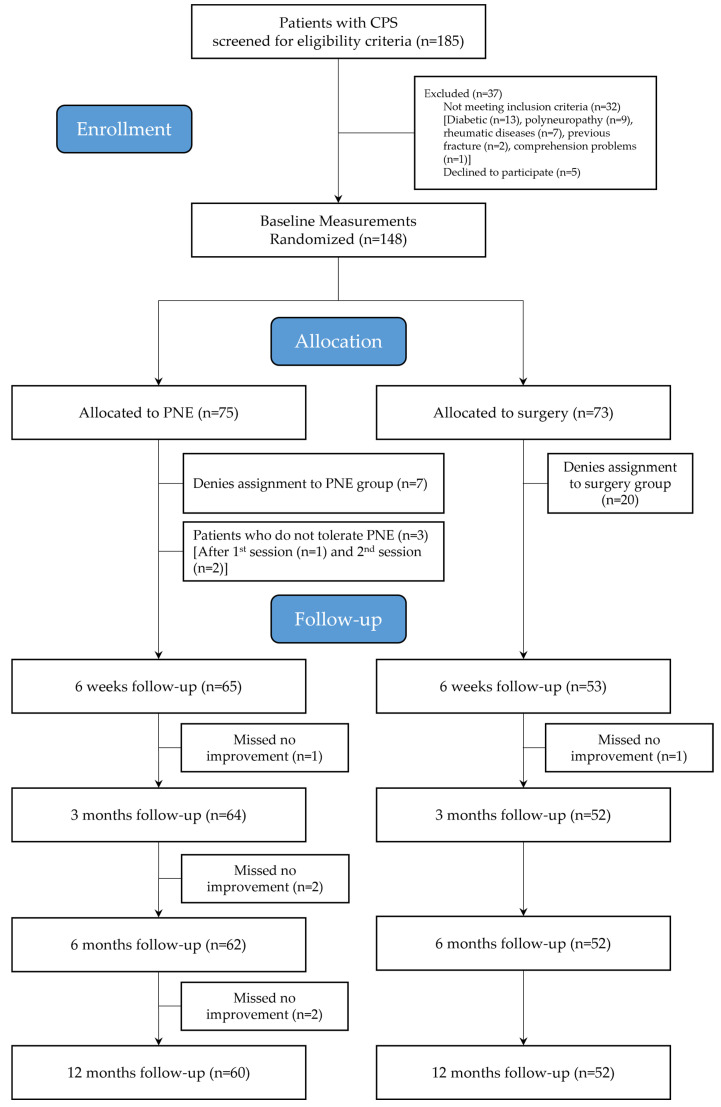
Flow diagram of patients throughout the course of the study.

Figure 2 shows the flow diagram of study participants. From a total of 185 patients admitted in the Traumatology Department during the study period, 148 met all the eligibility criteria and volunteered to participate. They were randomly assigned to receive either surgery (*n* = 73) or electrolysis (*n* = 75), although ultimately 112 patients (52 and 60 for surgery and electrolysis, respectively) completed all the follow-up assessments. Reasons for withdrawal are presented in Figure 2. At the time of randomization, most patients who did not receive the treatment were in the surgery group (20 [27.4%] versus 10 [13.3%] in the electrolysis group).

Overall, no between-group differences were observed at the baseline for most sociodemographic and clinical variables. However, patients in the electrolysis group presented lower baseline pain, despite a higher proportion having more severe involvement on the dominant side (Table 1). Differential attrition between groups at the end of follow-up was 8.7% (20% minus 28.7% in the electrolysis and surgery groups), respectively. No significant differences were found between participants who withdrew from the study and those who completed it. Additionally, withdrawals from both groups also showed no differences (Table 1) and no one patient’s characteristic was associated with withdrawals in the regression models.

### 3.1. Adverse Events

Table 2 shows the incidence of mild-to-very severe adverse events reported by the patients in the whole group and per patient in each group at six weeks of follow-up. The electrolysis group had only two adverse events, none of which were severe–very severe. The surgery group had a total of 100, with 17 being severe–very severe. The electrolysis group has a 98% and 99.9% lower incidence rate of mild–moderate and severe–very severe events, respectively, than the surgery group (i.e., IRR = 0.020 and 0.001).

### 3.2. Effectiveness and Patterns over Time

**Figure 3:**
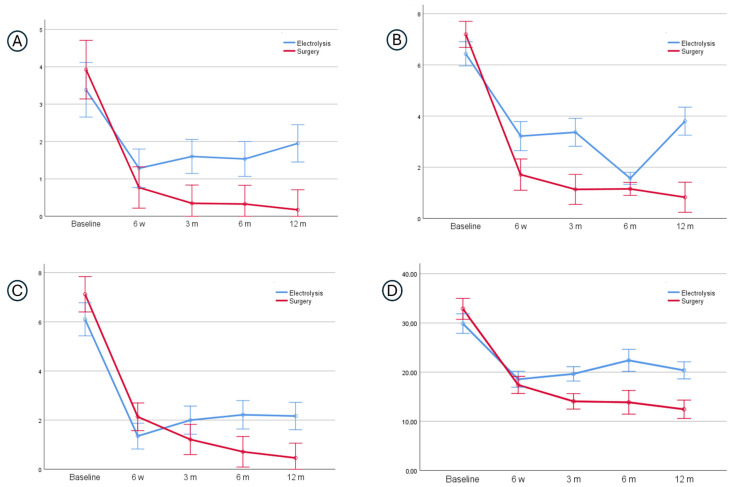
Pattern of improvement of n° nights (0–7) waking up due to symptoms (**A**), paresthesia (0–10) (**B**), pain (0–10) (**C**) and BCTQ-SSS Total (0–55) (**D**) at baseline and after 6 weeks and 3, 6, and 12 months of the intervention (electrolysis and surgery).

Table 3 and Figure 3 present the pattern of improvement of all baseline symptoms in both groups. The plotted trend lines show that symptom progression did not follow a smooth, continuous decline; rather, the patterns included periods of change (improvement) interspersed with phases of stability. Overall, the surgery group demonstrated an initial sharp reduction in the severity of the three symptoms (waking up, paresthesia and pain) as well as in the multidimensional symptom score (i.e., BCTQ-SSS) between baseline and 6 weeks, followed by a more gradual improvement up to the third month and then a plateau in symptom severity through to the 12-month follow-up. Furthermore, the trend lines for the electrolysis group also show an initial strong reduction in three symptoms until 6 weeks. However, no further improvement was observed until the third month. From that point onward, the number of nocturnal awakenings and pain levels remained stable through to the 12-month follow-up, whereas paresthesia showed a transient improvement at the 6-month mark.

The trend lines for both groups were non-parallel over time, and statistical analysis revealed a significant time–group interaction for all symptoms and their multidimensional score (Figure 3), indicating different patterns of improvement between the two groups. The surgery group showed patterns of improvement that were consistently better all times, and all between-group comparisons of mean scores—except for pain—showed statistically significant differences when averaged across the repeated measures (Table 3). Specifically, mean differences between groups were 0.84 (CI 99% = 0.32–1.37) for nights, 1.27 (CI 99% = 0.82–1.73) for paresthesia, 4.04 (CI 99% = 2.15–5.93) for BCTQ-SSS, and 0.44 (CI 99% = −0.12–1.0) for pain. Comparisons with multivariate analyses showed that adjustment for the baseline imbalances of potential prognostic indicators minimally influenced the results. Therefore, only the unadjusted analyses are presented. Additional sensitivity analyses found no impact from the duration of the current episode (*p* = 0.30) or location of the pain (*p* = 0.42) on the effect of surgery for the main primary outcome (i.e., nights waking up).

In both groups, the patterns of improvement for functioning (Table 3) were similar to those identified for pain. Statistical analysis also revealed a significant time–group interaction for them (F = 14.6, *p* = 0.001, ηp^2^ = 0.33), however, non-significant between-group differences (F = 4.02, *p* = 0.47, ηp^2^ = 0.03).

## 4. Discussion

This RCT compared the short-, medium-, and long-term outcomes of OCTR and PNE in patients with moderate-to-severe median nerve entrapment in the carpal tunnel, analyzing the pattern of improvement and symptom resolution. It also compared safety in the short term.

The number of adverse events in the group receiving surgery were similar to previous studies [22]. Our results showed that safety was higher in the electrolysis group compared to the surgery group. Moreover, it was very relevant that the electrolysis group had no severe adverse events. This was expected given the ultrasound-guided nature of the intervention. The absence of an infectious process was also expected in the electrolysis group due to the germicidal and bactericidal nature of the galvanic electric current [24].

Regarding effectiveness, the results show that both interventions significantly improved the patient’s symptoms, demonstrating a homogeneous pattern. In the short term (6 weeks), both interventions did not show significant differences; however, in the medium (3 months) and long term (6 and 12 months), surgery was slightly more effective.

Unlike treatments based on manual therapy, splints or corticosteroid injections, with effects mainly based on pain relief in the short term [6], surgical decompression and PNE directly address the structural cause of median nerve compression, resulting in long-term sustained pain relief, indicating their long-term effectiveness in addressing the underlying pathology of CTS.

The results of the present study in the surgery group were compatible with the improvement described in terms of symptoms and hand function by other authors using moderate-to-severe cases of CTS [22,25,26,27]. As expected, the results in the electrolysis group demonstrated a positive effect on median nerve release, supporting the fibrolytic effect previously described in animal models [13] and the promising outcomes reported in case series [14].

Furthermore, the findings of the present trial are consistent with the recommendations of the Cochrane review comparing surgical and non-surgical treatments for CTS, which highlights a balance between long-term effectiveness and potential risks associated with surgery [6]. In our study, OCTR demonstrated superior long-term reductions in symptom severity, particularly for nocturnal awakenings and paresthesia, supporting the evidence that surgery provides greater long-term clinical benefit. At the same time, ultrasound-guided PNE achieved sustained, clinically meaningful symptom improvement with a markedly lower incidence of adverse events. This pattern aligns with the Cochrane review’s conclusion that surgery should not be routinely recommended for all patients and that less invasive approaches may be appropriate in selected cases. Together, these results support a stepped-care approach in which treatment selection is guided by symptom severity, patient preferences, and risk–benefit considerations.

In our study, the differential withdrawal observed between treatment groups, particularly prior to intervention in the surgical arm, warrants careful consideration. This pattern likely reflects patient reluctance to undergo surgery, as willingness to accept carpal tunnel release is known to be influenced by patient expectations, symptom perception, and fear of surgery [28]. To explore the potential impact of attrition, baseline characteristics of participants who completed follow-up and those who withdrew were compared, and no clinically relevant differences were identified. In addition, regression analyses did not reveal any baseline variables associated with withdrawal. To further assess the robustness of the findings, covariate-adjusted and sensitivity analyses were conducted, yielding results consistent with the primary analyses.

### 4.1. Clinical Practice Implications

To date, surgery was the only evidence-based therapeutic option for cases with severe STC or when non-invasive options (e.g., physiotherapy, wrist splints) proved to be ineffective in the long term [7]. This clinical trial showed that PNE offers a sustained pattern of improvement over time (12-month follow-up). Based on these results, PNE may serve as an alternative to surgery as a first-line option for symptom management in moderate or severe STC and could be considered in several scenarios. In the first place, it can be used while patients await surgery, as waiting times for carpal tunnel release are often prolonged in clinical practice [22]. PNE offers a viable option for managing symptoms in the long term, with the potential to delay the need for surgery. Secondly, considering that our study and others [28] have shown that many patients do not accept or cancel surgical procedures during the waiting period, PNE could also be offered. Third, when symptoms persist after surgery, approximately 1–5% of patients require surgical revision [29,30]. In these cases, revision carpal tunnel release (CTR) is less successful than primary CTR because up to 40% of patients have unfavorable outcomes [31,32] and perineural adhesions or fascicular scarring are observed in 88% of patients undergoing revision surgery [32,33]. For this reason, it would be possible to use PNE due to its fibrolytic effect [13]. Fourth, when recurrent carpal tunnel syndrome occurs, which was defined as an asymptomatic period after intervention followed by a recurrence of symptoms [34], especially if the patient has underlying conditions such as diabetes, rheumatoid arthritis or tobacco use [35]. According to the results of this study, PNE could be repeated if symptoms are recurrent. Finally, many patients with CTS present and bilateral symptoms are candidates for simultaneous bilateral procedures [36]. In this case, surgery could be considered on the more-affected hand and PNE could be applied to the contralateral hand.

### 4.2. Study Limitations and Future Research Studies

The excellent results observed in the surgical and electrolysis groups may be partly attributable to the high level of expertise of the clinicians involved, each with more than 10 years of experience in their respective techniques. In addition to operator expertise, the use of a standardized and well-defined methodological protocol [20], including ultrasound guidance, consistent intervention parameters, and controlled follow-up assessments, likely played a relevant role in achieving the reported results. Consequently, these findings may not be fully generalizable to clinical settings lacking similar methodological rigor or operator training. Future studies should specifically examine whether comparable safety profiles and patterns of clinical improvement can be reproduced when these interventions are applied by less experienced clinicians or under different methodological conditions.

Electrodiagnostic tests, including nerve conduction studies (NCS) and needle electromyography (EMG), were not included as an outcome variable in this study. Although NCS and EMG are generally well tolerated by patients [37], our experience is that it is difficult to complete the follow-up records because patients describe a certain degree of discomfort or pain during these tests and they often refuse further evaluations due to their clinical improvement. Nevertheless, it is important to consider that although neurophysiological improvements occur post-surgery, full normalization may not be achieved in all patients [38,39].

In the electrolysis group, PNE included a protocol with a cycle of four sessions applied every 7 days with the aim of standardizing the procedure for all patients. Although this protocol has demonstrated a positive effect on symptom resolution, future studies should attempt to optimize outcomes regarding additional sessions during the follow-up period (short- and medium-term) or alongside other conservative treatments, like manual therapy, splinting and nerve-gliding exercises. Theoretically we could add the mechanical and neurophysiological effects of these techniques [25,40,41] with PNE, which directly addresses the structural cause of median nerve compression [19].

In the surgical group, the procedure consisted of open carpal tunnel release (OCTR). Future studies could compare PNE with ultrasound-guided surgical procedures [42,43] or endoscopic carpal tunnel release (ECTR) [44]. To date, no significant differences have been found between OCTR and ECTR in terms of symptom relief and overall complication rates [45,46,47]. However, ECTR was associated with a shorter time to return to work [46] and with a 2.96 times greater likelihood of requiring revision carpal tunnel release within one year [47] and being more cost-effective [48], compared to OCTR.

Additionally, it would be interesting to investigate whether surgery and PNE could provide more lasting effects after several years of follow-up. Long-term follow-up (>1-year) is particularly important in determining the recurrence of symptoms, the need for additional treatments, and the overall cost effectiveness and patient satisfaction of each intervention. Considering only the United States, over 600,000 carpal tunnel release procedures are performed annually, with an economic impact of at least $2 billion [49].

## 5. Conclusions

This RCT indicates that US-guided PNE is a safe, minimally invasive intervention associated with sustained improvements in symptoms and function in patients with moderate-to-severe CTS, with fewer adverse events than OCTR. Both interventions produced significant clinical improvement; however, OCTR demonstrated greater long-term reductions in symptom severity, indicating superior long-term effectiveness. Accordingly, PNE should not be considered equivalent or superior to surgery but may offer meaningful benefits as a first-line or bridging treatment in selected scenarios, including for patients unwilling to undertake or unsuitable for surgery or awaiting intervention.

## Figures and Tables

**Table 1:** Main characteristics by group.

Variables	Electrolysis		Surgery	
	All Randomized (*n* = 75)	Withdrawals (*n* = 15)	All Randomized (*n* = 73)	Withdrawals (*n* = 21)
Demographic and clinical				
Women, *n* (%)	58 (77.3)	9 (60.0)	48 (65.8)	12 (57.1)
Age (mean ± SD)	49.8 ± 12.2	51.9 ± 15.8	53.9 ± 13.8	57.2 ± 15.4
BMI (mean ± SD)	28.5 ± 5.5	29.3 ± 4.2	28.5 ± 4.5	28.8 ± 3.2
Duration of episode (mean ± SD)	30.8 ± 24	30.2 ± 26	32.2 ± 33	30.4 ± 33.8
Bilateral complaints, *n* (%)	57 (76)	13 (86.7)	50 (68.5)	13 (61.9)
Dominant side, *n* (%)	56 (74.7)	14 (93.3)	43 (58.9)	14 (66.7)
Previous episodes, *n* (%)	42 (56.8)	7 (46.7)	33 (45.8)	8 (38.1)
Sick leave, *n* (%)	4 (5.3)	2 (13.3)	4 (5.5)	2 (9.5)
Primary outcomes				
Nights waking up (0–7)				
Number (median, IQR)	3 (6)	4 (5)	3 (8)	4 (6)
People with 0 nights, *n* (%)	16 (21.3)	2 (13.3)	16 (21.9)	5 (23.8)
Paresthesia (0–10)				
Severity (median, IQR)	7 (3)	7 (3)	7 (3)	7 (3)
People with score < 2, *n* (%)	2 (2.7)	0 (0)	0 (0)	0 (0)
Pain (0–10)				
Severity (median, IQR)	6 (3)	6 (4)	8 (4)	6 (3)
People with score < 2, *n* (%)	7 (9.3)	2 (13.3)	4 (5.5)	2 (9.5)
Symptom severity (0–55)				
Severity (mean ± SD)	30.9 ± 7.7	35.1 ± 6.2	32.7 ± 8.3	32.3 ± 9.9
People with score < 2, *n* (%)	1 (1.3)	0 (0)	1 (1.4)	1 (2.8)
Secondary outcome				
Functional status score				
Severity (mean ± SD)	19.9 ± 6.0	21.8 ± 7.2	21.0 ± 6.7	19 ± 7.6

**Table 2:** Incidence of adverse events (AEs) by severity grading, type and treatment group.

	Electrolysis (*n* = 65)			Surgery (*n* = 53)		
	All	Mild–Moderate	Severe–Very Severe	All	Mild–Moderate	Severe–Very Severe
**Total AEs**	2	2	0	100	83	17
**AEs per patient**	0.03	0.03	0	1.89	1.57	0.32
IRR *	0.016	0.020	0.001	Reference *	Reference *	Reference *
**AEs per patient by type**						
Painful scar	0	0	0	0.754	0.660	0.094
Stiffness of the wrist	0	0	0	0.301	0.207	0.094
Wound infection	0	0	0	0.188	0.151	0.038
Alteration in trophism	0	0	0	0.188	0.151	0.038
Nerve damage	0.015	0.015	0	0.132	0.113	0.019
Tendon damage	0	0	0	0.038	0.038	0
Swelling of the wrist	0	0	0	0.188	0.189	0
Others	0.015	0.015	0	0.094	0.057	0.038

* IRR: Incidence rate ratio (AE per patient of electrolysis/AE per patient in surgery -reference-).

**Table 3:** Pattern of improvement of primary and secondary outcomes after 6 weeks and 3, 6, and 12 months.

Variables by Week/Month	Electrolysis (*n* = 60)	Surgery (*n* = 52)	Interaction Effect F-Value (*p*-Value); ηp^2^	Main Effects F-Value (*p*-Value); ηp^2^	
				Between ^a^ Subjects	Within ^b^ Subjects
**Primary outcomes**					
N° nights waking up due to symptoms (mean ± SD)					
6 w	1.3 ± 2.0	0.8 ± 2.0	4.187 (0.003); 0.135	10.076 (0.002) ^a^; 0.084 19.972 (0.000) ^b^; 0.427	
3 m	1.6 ± 2.2	0.4 ± 1.1			
6 m	1.5 ± 2.1	0.3 ± 1.4			
12 m	2.0 ± 2.5	0.2 ± 1.0			
Paresthesia during day (mean ± SD)					
6 w	3.2 ± 1.8	1.7 ± 2.6	18.357 (0.000); 0.4.7	232.833 (0.000) ^a^; 0.220 232.833 (0.000) ^b^; 0.897	
3 m	3.4 ± 2.4	1.1 ± 1.8			
6 m	1.6 ± 1.0	1.2 ± 0.8			
12 m	3.8 ± 2.5	0.8 ± 1.6			
Pain during day (mean ± SD)					
6 w	1.4 ± 1.8	2.13 ± 2.3	9.568 (0.000); 0.263	2.391 (0.125) ^a^; 0.021 92.694 (0.000) ^b^; 0.776	
3 m	2.0 ± 2.6	1.2 ± 1.7			
6 m	2.2 ± 2.9	0.7 ± 1.1			
12 m	2.2 ± 2.9	0.5 ± 0.9			
Symptoms severity score (mean ± SD)					
6 w	18.6 ± 6.1	17.4 ± 6.6	13.388 (0.000); 0.334	17.959 (0.000) ^a^; 0.140 94.753 (0.000) ^b^; 0.780	
3 m	19.7 ± 6.8	14.1 ± 4.2			
6 m	22.4 ± 10.7	13.9 ± 5.7			
12 m	20.4 ± 8.8	12.4 ± 3.0			
**Secondary outcomes**					
Functional status score (mean ± SD)					
6 w	14.6 ± 5.8	17.3 ± 8.3	14.579 (0.000); 0.353	4.024 (0.47) ^a^; 0.35 37.852 (0.000) ^b^; 0.586	
3 m	15.0 ± 5.8	12.9 ± 6.3			
6 m	15.3 ± 5.9	10.1 ± 3.9			
12 m	16.1 ± 7.0	10.2 ± 4.4			

^a^ Difference between subjects; ^b^ Difference within subjects.

## Data Availability

The data that support the findings of this study are available from the corresponding author upon reasonable request. The data are not publicly available due to confidentiality reasons.

## Abbreviations

The following abbreviations are used in this manuscript:

BCTQ-SSS	Boston Carpal Tunnel Questionnaire Symptom Severity Scale
BCTQ FSS	Boston Carpal Tunnel Questionnaire Functional Status Scale
CTS	Carpal Tunnel Syndrome
ECTR	Endoscopic Carpal Tunnel Release
EMG	Electromyography
NCS	Nerve Conduction Studies
OCTR	Open Carpal Tunnel Release
PNE	Percutaneous Needle Electrolysis
RCT	Randomized Controlled Trial
TCL	Transverse Carpal Ligament
